# Initiation of antidepressants in young adults after ischemic stroke: a registry-based follow-up study

**DOI:** 10.1007/s00415-021-10678-4

**Published:** 2021-06-24

**Authors:** Jenna Broman, Karoliina Aarnio, Anna But, Ivan Marinkovic, Jorge Rodríguez-Pardo, Markku Kaste, Turgut Tatlisumak, Jukka Putaala

**Affiliations:** 1 Department of Neurology, Helsinki University Hospital and University of Helsinki, Haartmaninkatu 4, 00029 Helsinki, Finland; 2 Department of Public Health, University of Helsinki, Helsinki, Finland; 3 Department of Neurology, La Paz University Hospital, Madrid, Spain; 4 Department of Clinical Neuroscience, Institute of Neuroscience and Physiology, Sahlgrenska Academy at University of Gothenburg, Gothenburg, Sweden; 5 Department of Neurology, Sahlgrenska University Hospital, Gothenburg, Sweden

**Keywords:** Antidepressants, Brain infarction, Stroke, Young adult

## Abstract

**Objective:**

Data on post-stroke use of antidepressants in young individuals are scarce. We examined pattern and factors associated with initiating post-stroke antidepressants (PSAD) after ischemic stroke (IS) in young adults.

**Methods:**

Helsinki Young Stroke Registry includes patients aged 15–49 years with first-ever IS, 1994–2007. Data on prescriptions, hospitalizations and death came from nationwide registers. We defined time of initiating PSAD as time of the first filled prescription for antidepressants within 1 year from IS. We assessed factors associated with initiating PSAD with multivariable Cox regression models, allowing for time-varying effects when appropriate.

**Results:**

We followed 888 patients, of which 206 (23.2%) initiated PSAD. Higher hazard of starting PSAD within the first 100 days appeared among patients with mild versus no limb paresis 2.53 (95% confidence interval 1.48–4.31) and during later follow-up among those with silent infarcts (2.04; 1.27–3.28), prior use of antidepressants (2.09; 1.26–3.46) and moderate versus mild stroke (2.06; 1.18–3.58). The relative difference in the hazard rate for moderate–severe limb paresis persisted both within the first 100 days (3.84, 2.12–6.97) and during later follow-up (4.54; 2.51–8.23). The hazard rate was higher throughout the follow-up among smokers (1.48; 1.11–1.97) as well as lower (1.78; 1.25–2.54) and upper white-collar workers (2.00; 1.24–3.23) compared to blue-collar workers.

**Conclusion:**

One-fourth of young adults started PSADs within 1 year from IS. We identified several specific clinical characteristics associated with PSAD initiation, highlighting their utility in assessing the risk of post-stroke depression during follow-up.

**Supplementary Information:**

The online version contains supplementary material available at 10.1007/s00415-021-10678-4.

## Introduction

Depression after stroke affects nearly a third of all patients, affecting the quality of life of the patient and worsening the outcome [1–6]. This appears to apply also to young individuals [7], thus remarkably affecting their general health, vocational performance, and family relations.

Most patients develop symptoms of depression shortly after the acute event [2, 6]. Disability and pre-stroke depression are the most consistently reported predictors of post-stroke depression in older patients, in addition to cognitive impairment, stroke severity, lack of social or family support, and anxiety [2, 6]. Similar factors are found to be associated with post-stroke depression in young stroke patients as well [8], although the evidence is scarce [8]. Post-stroke depression itself is associated with lower quality of life and reductions in activities of daily living, impaired functional recovery as well as increased disability and mortality [2, 6, 9–15]. Depression after stroke is associated with long-term mortality also in younger individuals [16] and even solely using antidepressants after stroke is suggested to increase mortality or lead to other unfavorable outcomes [9, 17, 18]. Moreover, stroke patients are treated with antidepressants more often than patients with other chronic conditions [19] while increasing stroke severity is shown to be associated with higher likelihood of newly prescribed antidepressant medication [20].

However, prevalence and factors associated with newly initiated post-stroke antidepressants (PSADs) are poorly known in the young. Thus, we aimed to characterize the initiation of antidepressants after ischemic stroke (IS) in young adults.

## Methods

### Study population

In this registry-based follow-up study, we studied patients with first-ever IS that occurred at the age of 15–49 years between January 1994 and May 2007. Our cohort originates from the Helsinki Young Stroke Registry (HYSR), consisting of 1008 consecutive young patients with first-ever IS treated in the Department of Neurology, Helsinki University Hospital, as identified from a prospective computerized hospital discharge database. We utilized the original WHO stroke definition, however also including those with imaging-positive findings of IS despite a short symptom duration [21]. We further combined HYSR data with the data from several national registries using personal identification number, which is assigned to every resident in Finland. From the present study, we excluded patients who had a false primary diagnosis, could not be linked to databases, died within 3 weeks from IS (early i.e., in-hospital deaths), or had antidepressant use within 1 year prior to index stroke (cannot be classified as initiating PSAD use during the follow-up).

### Baseline data

Baseline laboratory and other diagnostic tests have been previously fully described [22]. Brain imaging with computed tomography (CT) or magnetic resonance imaging (MRI) was performed for all patients. We collected patients’ sociodemographic data, i.e., age, sex, socioeconomic status [23], prior antidepressant use, prior psychiatric hospitalization; data on risk factors for IS, i.e., cigarette smoking status at the time of index IS, heavy alcohol use (consumption over 200 g a week), history of drug abuse, cardiovascular disease, atrial fibrillation, hypertension, dyslipidemia, diabetes mellitus type 1 and 2; and data on stroke-related variables measured at admission, i.e., NIH Stroke Scale (NIHSS), Trial of Org 10,172 in Acute Stroke Treatment (TOAST) classification, infarct size and laterality, silent infarcts, and leukoaraiosis [22].

Information on hospitalizations from any psychiatric reasons, including mood disorders, psychotic disorders, and other psychiatric reasons (International Classification of Diseases, eighth revision (ICD-8) categories 291–299, 300–301, ninth revision (ICD-9) categories 295–298, 300–301, 309, and tenth revision (ICD-10) categories F04–F09, F20–F48, F60–69) ever prior to IS was obtained from the Care Register for Health Care, maintained by the National Institute for Health and Welfare, Finland, from 1969 to the end of 2011. Socioeconomic status was classified according to the patient’s occupation as upper white-collar worker, lower white-collar worker, blue-collar worker, and other (entrepreneur, student, pensioner, and unemployed) or unknown [23]. Based on NIHSS score at admission, stroke severity was categorized as mild (NIHSS 0–6), moderate (NIHSS 7–14) and severe (NIHSS ≥ 15).

### Initiation of antidepressants

We obtained data on filled prescriptions (i.e., purchases) from 1993 to the end of 2011, including date of purchase and Anatomical Therapeutic Chemical (ATC) codes (WHO Collaborating Centre for Drug Statistics Methodology, 2018), from the Drug Prescription Register kept by the Social Insurance Institution of Finland. The Drug Prescription Register includes information on all prescriptions assigned by any physician in Finland. In Finland, prescribed medications are entitled to reimbursement scheme (reimbursement of 40–100% of the medicine’s price) and the Social Insurance Institution of Finland can only reimburse a quantity of medication equivalent to 3 months of treatment at any one time, thus the typical interval of the purchases are 3 months. Based on ATC code N06A, we identified those who purchased antidepressants before and after the index IS. In this study, we defined PSAD initiators to be those patients who had at least one purchased antidepressant medication prescription during the first year after the index IS. We considered patients with filled prescription earlier than 1 year prior to index IS as prior antidepressant users. The follow-up started at the index stroke and ended at the first PSAD purchase, death, or at 1 year from IS, whichever came first.

### Statistical analyses

The baseline characteristics of the patients are presented with descriptive statistics for all IS patients and stratified by initiation of PSAD. We reported median and interquartile range for continuous variables and number of observations and percentages for categorical variables. The missingness of data was reported as number of observations and percentages. We demonstrated the numbers of different types of antidepressants used by Venn diagram.

Univariate and multivariable Cox proportional hazard’s regression models were fitted to the data on the entire study population to analyze the hazard of initiating antidepressants within the first year after index IS with regard to the baseline characteristics. Based on the univariate Cox regression model, we identified variables with *p* value < 0.10 to be further studied in the multivariable analysis. In multivariable analysis, we considered age and sex as essential to control for and forced them into the model. The final model was constructed using backward stepwise selection with cut-off of *p* < 0.05 for variables to be included. There was no considerable collinearity (variance inflation factors > 5) between covariates included into the final model. We checked whether proportional hazards assumption held for the variables included into the final Cox regression model. In case of non-proportional hazards, we allowed for time-varying coefficients. From these analyses, we reported crude and adjusted as well as time-varying hazard ratios and their 95% confidence intervals (CI) for the initiation of antidepressants. In addition, we modeled and plotted the hazard rate of initiating PSAD as a function of follow-up time using the restricted cubic spline function. The cumulative incidence curves assessing differences in survival with log-rank test were used to visualize the overall and stratum-specific initiating of PSAD. We did additional analysis by also including patients with antidepressant use within 1 year prior to IS to see whether the main results would change. We used Kaplan–Meier curves to examine the survival probabilities for patients using antidepressants at different time points before the index event.

Statistical analyses were performed with SPSS 25.0 for Windows (SPSS Inc., IBM, Armonk, NY, USA) and with the R software (R Core Team, 2020) [24].

## Results

### Initiation of antidepressants after stroke

**Fig. 1:**
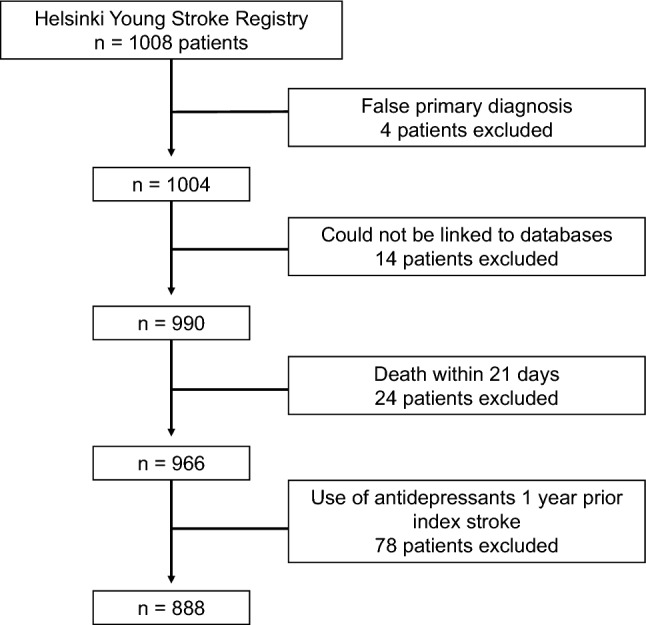
Flow chart of the number of patients included in the study

After applying exclusion criteria, a total of 888 patients were followed up (Fig. 1), of which 206 (23.2%) initiated PSAD in the first year after index IS. During the first year from IS, 14 (1.6%) patients without purchase of antidepressants and 3 (0.3%) patients with PSAD died. A total of 73 (8.2%) patients and 31 (15.0%) of the PSAD initiators had used antidepressants at some point earlier than 1 year preceding the index IS.

**Fig. 2:**
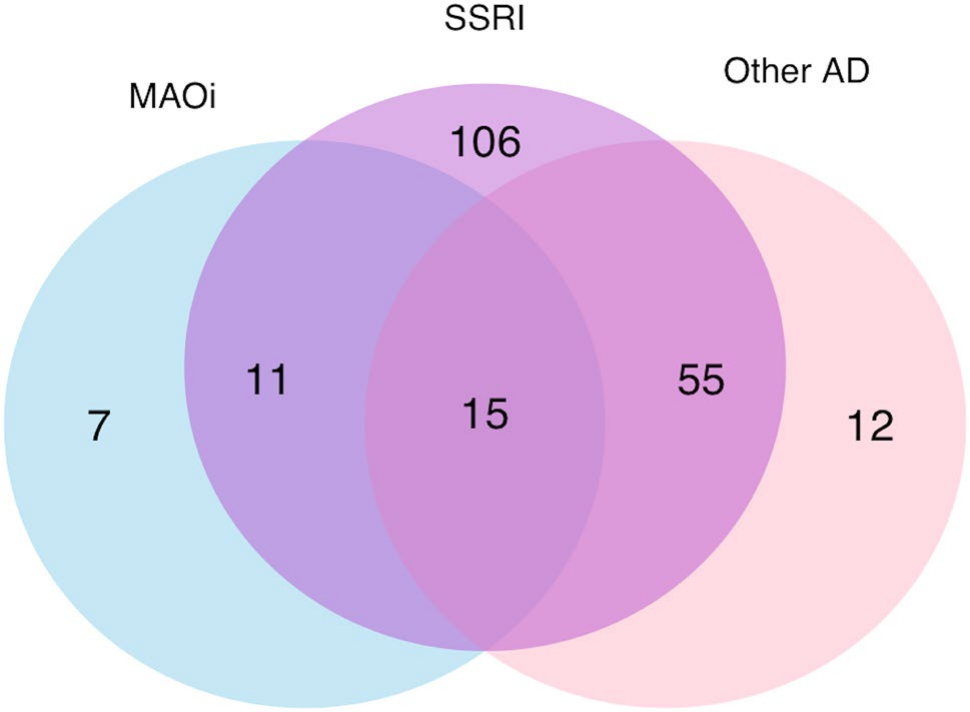
Venn diagram and numbers of different antidepressants initiated within the first year after index ischemic stroke. *MAOi* non-selective monoamine reuptake inhibitor, *SSRI* selective serotonin reuptake inhibitor, *AD* antidepressant

Of the 206 patients initiating PSAD, 33 (16.0%) used non-selective monoamine reuptake inhibitors, 187 (90.8%) used selective serotonin reuptake inhibitors (SSRIs), and 82 (39.8%) used some other antidepressant, with considerable overlap and use of different antidepressants over time (Fig. 2).

**Fig. 3:**
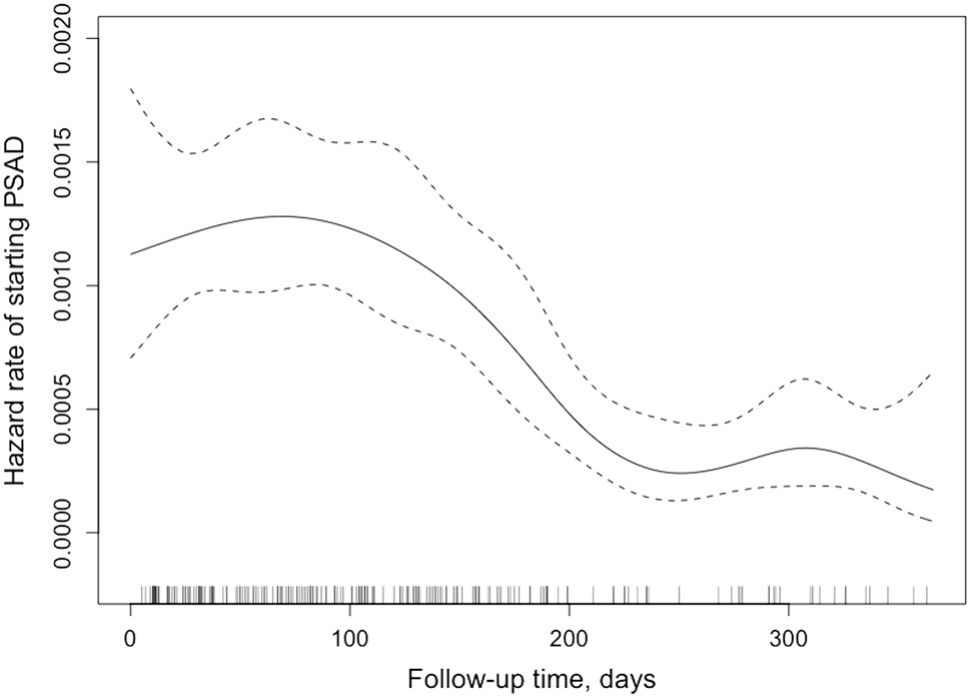
Hazard rate curve with 95% confidence interval of starting post-stroke antidepressants (PSAD)

Median time to first PSAD purchase was 102 days (interquartile range 50–163) and the hazard rate of initiating PSAD was highest during the first 100 days since IS (Fig. 3). A total of 101 (49.0%) of the PSAD initiators started use within 100 days and 105 (51.0%) after 100 days from IS. The only statistically significant difference in clinical characteristics between the early and late PSAD starters was the more frequent history of psychiatric hospitalizations among late starters (data not shown). A total of 116 (56.3%) PSAD initiators had their second purchase within 3 months and 159 (77.2%) within 6 months from the first filled prescription. Eleven (11.7%) of patients under 30 years old, 47 (25.0%) of patients aged 30–39 years and 148 (24.4%) of patients over 40 years started PSAD*.* However, age was not statistically significant factor in the multivariable models.

Table 1 depicts the baseline characteristics of PSAD non-initiators and initiators (Supplementary Material 1 presents the numbers for these groups combined). Compared to patients without starting PSAD, PSAD starters were less often blue-collar workers, more often cigarette smokers, they had more often moderate and severe strokes and silent infarcts, as well as large anterior infarcts and infarcts caused by large-artery atherosclerosis. At the time of hospital discharge, PSAD starters had more often limb paresis and aphasia. These patients also had more often history of psychiatric hospitalization and antidepressant use.

**Table 1:** Baseline characteristics of those not initiated and initiated post-stroke antidepressants within the first year after index IS

Characteristic	Not initiated PSAD 682 (76.8%)	Initiated PSAD 206 (23.2%)
	*n* (%) or median (interquartile range)	
Sociodemographic variables		
Age at IS, years	43.0 (37.0–47.0)	44.5 (38.0–47.0)
Male sex	440 (64.5)	124 (60.2)
Socioeconomic status^a^		
Upper white-collar worker	76 (11.4)	25 (12.3)
Lower white-collar worker	157 (23.5)	61 (30.0)
Blue collar worker	300 (44.9)	69 (34.0)
Other/unknown	135 (20.2)	48 (23.6)
Prior antidepressant use	42 (6.2)	31 (15.0)
Psychiatric hospitalization prior to IS	32 (4.7)	16 (7.8)
Risk factors for IS		
Atrial fibrillation	26 (3.8)	8 (3.9)
Cardiovascular disease	62 (9.1)	23 (11.2)
Diabetes mellitus type 1	30 (4.4)	10 (4.9)
Diabetes mellitus type 2	43 (6.3)	12 (5.8)
Dyslipidemia	403 (59.1)	125 (60.7)
Hypertension	265 (38.9)	91 (44.2)
Current cigarette smoking	286 (41.9)	101 (49.0)
Heavy alcohol use	84 (12.3)	28 (13.6)
History of drug abuse	15 (2.2)	6 (2.9)
Stroke-related variables measured at hospital admission		
Silent infarcts	76 (11.1)	37 (18.0)
Leukoaraiosis	34 (5.0)	14 (6.8)
Infarct size		
Small	317 (46.5)	77 (37.4)
Medium	202 (29.6)	48 (23.3)
Large anterior	75 (11.0)	60 (29.1)
Large posterior	88 (12.9)	21 (10.2)
Laterality^b^		
Right	292 (44.6)	87 (43.5)
Left	303 (46.3)	92 (46.0)
Both	60 (9.2)	21 (10.5)
TOAST		
Large-artery atherosclerosis	40 (5.9)	24 (11.7)
Cardioembolism	128 (18.8)	35 (17.0)
Small-vessel disease	105 (15.4)	22 (10.7)
Other	171 (25.1)	62 (30.1)
Undetermined causes	238 (34.9)	63 (30.6)
NIHSS at admission		
0–6, mild	566 (83.0)	110 (53.4)
7–14, moderate	79 (11.6)	61 (29.6)
≥ 15, severe	37 (5.4)	35 (17.0)
Disability at discharge		
Limb paresis at discharge^c^		
No	528 (78.3)	94 (45.6)
Mild	87 (12.9)	37 (18.0)
Moderate–severe	59 (8.8)	75 (36.4)
Aphasia at discharge^c^	122 (18.1)	71 (34.5)

*PSAD* post-stroke antidepressant, *IS* ischemic stroke, *NIHSS* NIH Stroke Scale, *TOAST* trial of org 10,172 in acute stroke treatment

^a^Data missing and not included in 14 (2.1%) PSAD non-initiators and 3 (1.5%) initiators

^b^Data missing and not included in 27 (4.0%) PSAD non-initiators and 6 (3.0%) initiators

^c^Data missing and not included in 8 (1.2%) PSAD non-initiators

In the univariate Cox regression analysis, several factors, including sociodemographic factors, risk factors for IS and clinical IS data, were associated with initiating PSAD (Table 2).

**Table 2:** Crude and adjusted numbers in univariate and multivariable Cox regression analyses with fixed coefficients for initiating antidepressants within the first year after index IS

Characteristic	Univariate analysis (*n* = 888 ^a^)	Multivariable analysis (*n* = 863)
	HR (95% CI)	aHR (95% CI)
Sociodemographic variables		
Age at IS, years	1.02 (1.00–1.04)	1.01 (0.99–1.03)
Male sex	0.83 (0.63–1.09)	0.85 (0.63–1.13)
Socioeconomic status		
Blue collar worker	Ref	Ref
Lower white-collar worker	1.55 (1.10–2.19)	1.75 (1.23–2.49)
Upper white-collar worker	1.36 (0.90–2.14)	1.99 (1.23–3.21)
Other/Unknown	1.47 (1.02–2.12)	1.41 (0.97–2.04)
Prior antidepressant use	2.31 (1.57–3.39)	1.47 (0.99–2.19)
Psychiatric hospitalization prior to IS	1.58 (0.95–2.63)	–
Risk factors for IS		
Atrial fibrillation	1.06 (0.52–2.15)	–
Cardiovascular disease	1.17 (0.76–1.80)	–
Diabetes mellitus type 1	1.09 (0.58–2.05)	–
Diabetes mellitus type 2	0.97 (0.54–1.74)	–
Dyslipidemia	1.04 (0.79–1.38)	–
Hypertension	1.22 (0.93–1.60)	–
Current cigarette smoking	1.26 (0.96–1.66)	1.49 (1.12–1.99)
Heavy alcohol use	1.07 (0.72–1.59)	–
History of drug abuse	1.31 (0.58–2.96)	–
Stroke-related variables measured at hospital admission		
NIHSS at admission		
0–6, mild	Ref	Ref
7–14, moderate	3.28 (2.39–4.48)	1.70 (1.15–2.49)
≥ 15, severe	3.73 (2.55–5.46)	1.22 (0.74–2.02)
Silent infarcts	1.60 (1.12–2.29)	1.49 (1.03–2.16)
Leukoaraiosis	1.37 (0.80–2.36)	–
Infarct size		
Small	Ref	
Medium	0.97 (0.67–1.38)	–
Large anterior	2.67 (1.90–3.74)	–
Large posterior	0.95 (0.87–1.53)	–
Laterality		
Right	Ref	–
Left	1.01 (0.75–1.35)	–
Both	1.16 (0.72–1.88)	–
TOAST		
Large-artery atherosclerosis	1.90 (1.19–3.04)	
Cardioembolism	1.03 (0.68–1.56)	–
Small-vessel disease	0.83 (0.51–1.34)	–
Other	1.37 (0.96–1.95)	–
Undetermined causes	Ref	
Disability at discharge		
Limb paresis at discharge		
No	Ref	
Mild	2.23 (1.52–3.26)	1.93 (1.28–2.91)
Moderate–severe	5.17 (3.81–7.02)	4.16 (2.72–6.36)
Aphasia at discharge	2.13 (1.60–2.84)	–

*HR* hazard ratio, *aHR* adjusted hazard ratio, *CI* confidence interval, *IS* ischemic stroke, *NIHSS* NIH Stroke Scale, *TOAST* trial of org 10,172 in acute stroke treatment, *Ref* reference

^a^*n* = 871 for socioeconomic status, *n* = 855 for laterality and *n* = 880 for limb paresis and aphasia in univariate analysis

The final multivariable model (*n* = 863, 2.8% excluded due to missing value in socioeconomic status and limb paresis at discharge) included age, sex, socioeconomic status, antidepressant use (earlier than 1 year) prior to stroke, current cigarette smoking at the time of index IS, NIHSS score at admission, limb paresis at discharge, and silent infarcts (Table 2). Cumulative incidence curves of initiating PSAD and numbers of patients at risk for all patients and for variables included to the final model are shown in Supplementary Material 2.

The proportional hazards assumption was violated for prior use of antidepressants, silent infarcts, stroke severity, and limb paresis at discharge. To account for non-proportionality, we split the follow-up time into two time strata (PSAD started ≤ 100 or > 100 days) and fitted the Cox regression model when allowing for time-varying coefficients (Table 3). Based on this model, moderate stroke symptoms on admission, limb paresis at discharge, higher socioeconomic status, prior use of antidepressants, silent infarcts, and current smoking were associated with a higher hazard rate of initiating PSAD. In addition, the model showed that prior use of antidepressants, moderate stroke symptoms at admission as well as silent infarcts, were only associated with PSAD initiated after 100 days. Moreover, mild limb paresis at discharge was only associated with PSAD initiated within the first 100 days and the relative difference in the hazard rate for moderate–severe limb paresis compared to no paresis persisted both within the first 100 days and during later follow-up.

**Table 3:** Adjusted numbers in multivariable Cox regression model with time-varying coefficients for initiating antidepressants within the first year after IS (*n* = 863, 2.8% excluded due to missing value in socioeconomic status and limb paresis at discharge)

Characteristic	Overall	Time-varying coefficients	
		PSAD started ≤ 100 days	PSAD started > 100 days
	aHR (95% CI)	aHR (95% CI)	aHR (95% CI)
Age at IS, years	1.01 (0.99–1.03)		
Male sex	0.84 (0.62–1.13)		
Socioeconomic status			
Blue collar worker	Ref		
Lower white-collar worker	1.78 (1.25–2.54)		
Upper white-collar worker	2.00 (1.24–3.23)		
Other/Unknown	1.44 (0.99–2.08)		
Prior antidepressant use		0.90 (0.47–1.75)	2.09 (1.26–3.46)
Current cigarette smoking	1.48 (1.11–1.97)		
NIHSS at admission			
0–6, mild		Ref	Ref
7–14, moderate		1.43 (0.84–2.42)	2.06 (1.18–3.58)
≥ 15, severe		0.77 (0.36–1.64)	1.79 (0.90–3.55)
Silent infarcts		0.96 (0.53–1.73)	2.04 (1.27–3.28)
Limb paresis at discharge			
No		Ref	Ref
Mild		2.53 (1.48–4.31)	1.34 (0.70–2.58)
Moderate–severe		3.84 (2.12–6.97)	4.54 (2.51–8.23)

The time strata used in case of time-varying coefficient are PSAD started ≤ 100 or > 100 days after index IS

*PSAD* post-stroke antidepressant, *aHR* adjusted hazard ratio, *CI* confidence interval, *IS* ischemic stroke, *NIHSS* NIH Stroke Scale, *Ref* reference

If also including patients with recent antidepressant use (*n* = 78) in the study population, higher socioeconomic status, antidepressant use before IS, silent infarcts, current smoking, and moderate to severe limb paresis at discharge were associated with increased hazard for starting PSAD. Mild limb paresis at discharge was associated with PSAD initiated within the first 100 days from IS, whereas moderate as well as severe stroke symptoms at admission were associated with PSAD initiated after 100 days (data not shown). In visual comparison, the cumulative survival probabilities were substantially different in patients with recent antidepressant use prior IS compared to those with earlier or no use prior IS (Supplementary Material 3). Furthermore, when combined on our analyses and adjusted also for other factors, antidepressant use within 1 year prior IS increased the hazard of starting PSAD (data not shown), suggesting these patients being those continuing their pre-stroke antidepressant use.

## Discussion

In this registry-based follow-up study on 888 young patients with first-ever IS, every fourth purchased antidepressants within the first year after stroke, mostly SSRIs. Moderate strokes compared with mild ones, limb paresis at discharge, silent infarcts, higher socioeconomic status, history of earlier antidepressant use, and current smoking increased the hazard for initiating PSAD.

The data especially on antidepressant use in young IS survivors are limited. In our study, the percentage of PSAD starters within the first year from IS (23%) corresponds with the post-stroke depression rates reported in previous studies including patients of any age, mainly older patients [1–4]. In the present study, median time to the first PSAD purchase from IS was around 3 months and SSRIs were the most frequently initiated antidepressants. Furthermore, those starting PSAD were more often older. Correspondingly, a large Danish cohort study found that stroke patients had a higher incidence of depression during the first 3 months after hospitalization compared with the reference population and that older age was significant risk factor for depression in both populations [6]. Similarly, in a recent study on 5070 consecutive first-ever ischemic stroke patients with a mean age of over 70 years [20], almost half of the previously untreated patients started antidepressant medication shortly after IS, the cumulative incidence of antidepressant treatment over 6 months post-stroke was 35%, and the most commonly prescribed antidepressants were SSRIs.

Furthermore, in that study [20], increasing stroke severity was associated with a higher likelihood of newly prescribed antidepressant after stroke. Association between stroke severity and initiating PSAD was also found in young individuals in our study. Those with moderate stroke symptoms at admission had higher hazard of starting PSADs compared to mild strokes. In addition, severe symptoms were associated with PSAD initiation univariately. However, this association was likely to be related to symptoms of limb paresis at hospital discharge, since 85% of those with severe stroke symptoms at admission had also limb paresis of some stage at discharge. Our study is in accordance with earlier findings that greater disability is associated with post-stroke depression not only in the general stroke population but also in younger individuals [2, 8, 25].

Silent infarcts and current smoking predicted the initiation of PSAD in our study, both of which can be indicators of poor lifestyle habits and poor adherence to self-care [26]. In accordance with our observations, one study found an association between silent lacunar infarction in basal ganglia and higher risk of depression [27]. Smoking is known to be associated with increased risk of depression in general [28, 29] and association between smoking and increased risk of post-stroke depression was reported recently [6, 30]. Furthermore, current smokers had higher NIHSS scores on admission in IS patients with small-vessel occlusions [31], but in our study, the frequencies of more severe strokes at admission did not differ significantly between smokers and non-smokers (data not shown). Heavy alcohol use was not associated with the initiation of PSAD in our study with relatively few heavy drinkers (*n* = 28, 13.6%) among patients initiating PSAD, but nor was it in a recent registry-based cohort study [6]. However, in these previous studies again, the age distribution is older compared to our study [6, 27, 30, 31]. The association between higher socioeconomic status and initiating PSAD in the present study might reflect a better compliance for starting antidepressant treatment. Expenses of the medicines are unlikely to be a significant reason not to purchase prescribed antidepressants among patients with lower socioeconomic status, since the costs are covered by the reimbursement system.

Our study has numerous strengths. It has a large study population of young patients with IS, detailed information on baseline stroke characteristics as well as other clinically important factors available. As an observational study, it brings the benefit of real-life setting compared to clinical trials. Data were collected from obligatory records required by health authorities, which reduces information bias. To assess the initiation of antidepressants, we excluded patients who had filled antidepressant prescriptions within 1 year prior to index stroke, since these patients were most likely continuing pre-stroke antidepressant use.

Also, we included only patients surviving the primary hospitalization period after IS and thus excluded 24 patients dying within the first 21 days. All of these patients died during the hospitalization, most of them (*n* = 20) within the first week after IS. Despite the fact that we had no information on actual compliance for antidepressant use, our study is likely to provide a good reflection of actual drug use, since our data consisted of only filled prescriptions and more than a half of the PSAD initiators had their second purchase within 3 months and over 3 of 4 initiators within 6 months, suggesting that those patients were also likely to become longer-term users. Furthermore, the present study has several methodological and analytical advantages, including a thorough assessment of the proportional hazards assumption and evaluation of the variation of both the ratio and rate of hazard of initiating PSAD over time.

Our study also has some inevitable limitations, and some sources of bias are challenging to control entirely due to its retrospective observational nature. It is noteworthy that the evidence on the value of the antidepressants for post-stroke depression and the range of different antidepressants available have remarkably changed over the long time period that our study covers. This presents some challenges in interpreting the results of the study and generalizing them when considering PSAD initiation occurred after this study. However, there were no consistent variations by calendar in initiation of PSAD (data not shown) and it is likely that the same factors are associated with PSAD initiation, since their association is not dependent on calendar time. This study assessed post-stroke (i.e., most likely stroke-related) initiation of antidepressants and accounted only PSAD started within the first year after IS. Although the data on antidepressant purchases prior to index IS did not reach up to 5 or more years in all patients, we estimated that approximately only 3 patients (9% of the previous antidepressant users) were potentially misclassified as a non-previous user. Even though the incidence of depression is still reported to be almost twice as high during the second year after stroke compared to reference population [6], the definition of starting PSAD applied in this study is relevant with respect to the research question on the post-stroke initiation of antidepressants. Despite it cannot be ruled out that PSAD started later than the first year after IS might be stroke-related in some individuals, by including also patients starting antidepressants after one or more years since IS death will most likely become a competing risk, unlike with our current method when the mortality rate within the first year after IS is low (*n* = 17, 1.9%). Furthermore, by focusing on PSAD initiated within the first year after IS, the results might be better utilized for post-stroke rehabilitation and follow-up. In our study population, 63% of PSAD initiators and 47% of non-initiators were discharged to institutional care or rehabilitation. We had no data on duration of the period of institutional rehabilitation and on the medication used during this time. However, institutional long-time hospitalization is less common in younger stroke patients compared to older population [32]. It is possible that some of those discharged to institutional rehabilitation started PSAD during this period and continued or stopped the use after institutional rehabilitation. The results form multivariable regression model including institutional rehabilitation suggested that the pattern of initiating PSAD was not affected by institutional rehabilitation (data not shown). Since the definition of initiating antidepressant use was based on filled prescriptions, we were not able to distinguish whether the indication for starting PSAD was depression, or some other reason, for example, to enhance neuroplasticity and promote stroke recovery. It is also possible that a patient diagnosed with depression had poor adherence to treatment and for this reason did not have any filled prescriptions. In addition, some possibly important risk factors for PSAD, such as diabetes [6], are relatively rare in younger population and thus the statistical power regarding these factors is limited.

In conclusion, even though additional research on young IS patients with larger study population, as well as prospective follow-up studies on post-stroke depression is needed to further assess the nature of our findings, we can conclude that previous history of depression as well as specific clinical characteristics are to be thoroughly assessed and post-stroke depression kept in mind in post-stroke rehabilitation and follow-up visits.

## Supplementary Information

Below is the link to the electronic supplementary material.

Supplementary file1 (DOCX 29 KB)

Supplementary file2 (DOCX 258 KB)

Supplementary file3 (DOCX 51 KB)

## Data Availability

We have documented the data, methods, and materials used to conduct the research in this report. The individual patient data are not publicly available because of legal restrictions.

## Code availability

The syntax of the study analyses may be available from the corresponding author upon reasonable request.
