# Supplementary material for: Initiation of antidepressants in young adults after ischemic stroke: a registry-based follow-up study
Source: J Neurol. 2021 Jun 24;269(2):956–65. doi: 10.1007/s00415-021-10678-4 (PMC8782780; doi:10.1007/s00415-021-10678-4)
Supplement: Supplementary file 1 — Supplementary file1 (DOCX 29 KB) [file 415_2021_10678_MOESM1_ESM.docx]

**Article Title:** Initiation of antidepressants in young adults after ischemic stroke – a registry-based follow-up study

**Journal:** Journal of Neurology

**Authors:** Jenna Broman, MD; Karoliina Aarnio, MD, PhD; Anna But, MSc, PhD; Ivan Marinkovic MD, PhD; Jorge Rodríguez-Pardo, MD, PhD; Markku Kaste MD, PhD; Turgut Tatlisumak MD, PhD; Jukka Putaala MD, PhD

**Corresponding author**

Jenna Broman

Department of Neurology, Helsinki University Hospital

Haartmaninkatu 4, FI-00029, Helsinki, Finland.

Tel: +358 9 4711

E-mail: [jenna.broman@fimnet.fi](mailto:jenna.broman@fimnet.fi)

**Online Resource 1** Baseline characteristics of IS patients included in the analyses

| Characteristic | Total  n = 888 |
| --- | --- |
| Sociodemographic variables |  |
| Age at IS, years | 44 (37–47) |
| Male sex | 564 (63.5) |
| Socioeconomic status ^a^ |  |
| Upper white-collar worker | 101 (11.4) |
| Lower white-collar worker | 218 (24.5) |
| Blue collar worker | 369 (41.6) |
| Other / Unknown | 183 (20.6) |
| Prior antidepressant use | 73 (8.2) |
| Psychiatric hospitalization prior to IS | 48 (5.4) |
| Risk factors for IS |  |
| Atrial fibrillation | 34 (3.8) |
| Cardiovascular disease | 85 (9.6) |
| Diabetes mellitus type 1 | 40 (4.5) |
| Diabetes mellitus type 2 | 55 (6.2) |
| Dyslipidemia | 528 (59.5) |
| Hypertension | 356 (40.1) |
| Current cigarette smoking | 387 (43.6) |
| Heavy alcohol use | 112 (12.6) |
| History of drug abuse | 21 (2.4) |
| Stroke-related variables measured at hospital admission |  |
| NIHSS at admission |  |
| 0–6, mild | 676 (76.1) |
| 7–14, moderate | 140 (15.8) |
| ≥15, severe | 72 (8.1) |
| Silent infarcts | 113 (12.7) |
| Leukoaraiosis | 48 (5.4) |
| Infarct size |  |
| Small | 394 (44.4) |
| Medium | 250 (28.2) |
| Large anterior | 135 (15.2) |
| Large posterior | 109 (12.3) |
| Laterality ^b^ |  |
| Right | 379 (42.7) |
| Left | 395 (44.5) |
| Both | 81 (9.1) |
| TOAST |  |
| Large-artery atherosclerosis | 64 (7.2) |
| Cardioembolism | 163 (18.4) |
| Small-vessel disease | 127 (14.3) |
| Other | 233 (26.2) |
| Undetermined causes | 301 (33.9) |
| Disability at discharge |  |
| Limb paresis at discharge ^c^ |  |
| No | 622 (70.7) |
| Mild | 124 (14.1) |
| Moderate–severe | 134 (15.2) |
| Aphasia at discharge ^c^ | 193 (21.9) |

IS = ischemic stroke; NIHSS = NIH Stroke Scale; TOAST = Trial of Org 10172 in Acute Stroke Treatment. Data are n (%) or median (interquartile range) ^a^ Data missing and not included in 17 (1.9%) patients. ^b^ Data missing and not included in 33 (3.7%) patients. ^c^ Data missing and not included in 8 (0.9%) patients.
