# Supplementary material for: Initiation of antidepressants in young adults after ischemic stroke: a registry-based follow-up study
Source: J Neurol. 2021 Jun 24;269(2):956–65. doi: 10.1007/s00415-021-10678-4 (PMC8782780; doi:10.1007/s00415-021-10678-4)
Supplement: Supplementary file 2 — Supplementary file2 (DOCX 258 KB) [file 415_2021_10678_MOESM2_ESM.docx]

**Article Title:** Initiation of antidepressants in young adults after ischemic stroke – a registry-based follow-up study

**Journal:** Journal of Neurology

**Authors:** Jenna Broman, MD; Karoliina Aarnio, MD, PhD; Anna But, MSc, PhD; Ivan Marinkovic MD, PhD; Jorge Rodríguez-Pardo, MD, PhD; Markku Kaste MD, PhD; Turgut Tatlisumak MD, PhD; Jukka Putaala MD, PhD

**Corresponding author**

Jenna Broman

Department of Neurology, Helsinki University Hospital

Haartmaninkatu 4, FI-00029, Helsinki, Finland.

Tel: +358 9 4711

E-mail: [jenna.broman@fimnet.fi](mailto:jenna.broman@fimnet.fi)

This Online Resource 2 figure was created with the R software (R Core Team, 2020) and Microsoft PowerPoint

**Online Resource 2** Cumulative incidence curves of initiating post-stroke antidepressants and numbers of patients at risk for (a) all patients and stratified by (b) socioeconomic status, (c) prior use of antidepressants, (d) current smoking, (e) NIH Score Scale at admission, (f) presence of silent infarcts, and (g) limb paresis at discharge


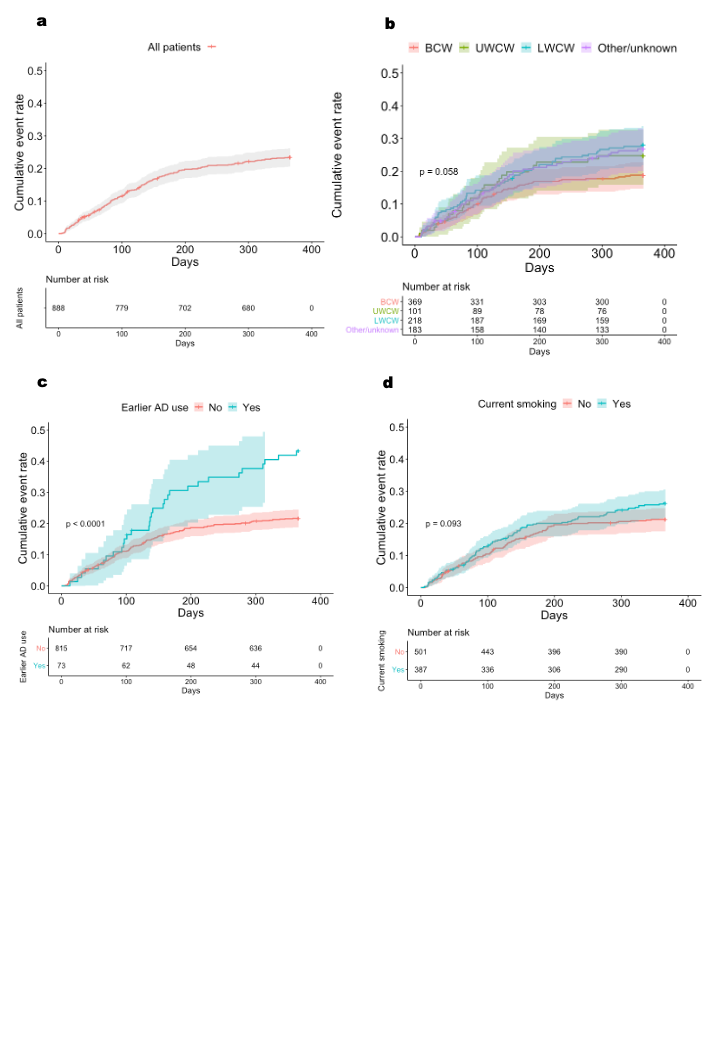


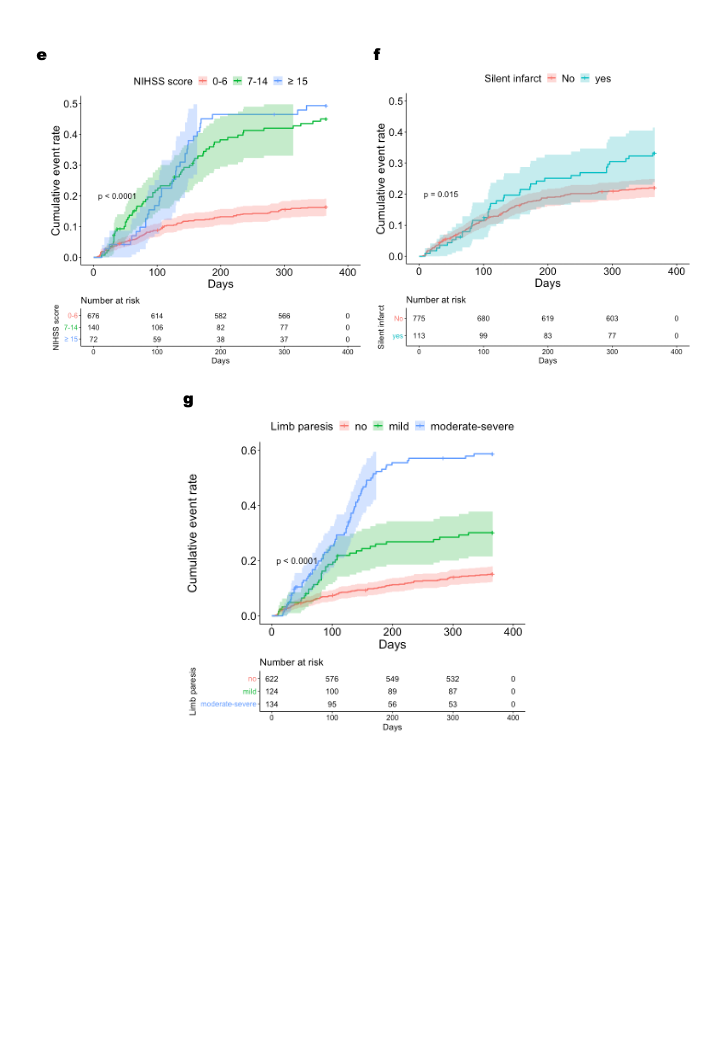


BCW = blue collar worker, UWCW = upper white-collar worker, LWCW = lower white-collar worker, AD = antidepressant, NIHSS = NIH Stroke Scale
