# Supplementary material for: Initiation of antidepressants in young adults after ischemic stroke: a registry-based follow-up study
Source: J Neurol. 2021 Jun 24;269(2):956–65. doi: 10.1007/s00415-021-10678-4 (PMC8782780; doi:10.1007/s00415-021-10678-4)
Supplement: Supplementary file 3 — Supplementary file3 (DOCX 51 KB) [file 415_2021_10678_MOESM3_ESM.docx]

**Article Title:** Initiation of antidepressants in young adults after ischemic stroke – a registry-based follow-up study

**Journal:** Journal of Neurology

**Authors:** Jenna Broman, MD; Karoliina Aarnio, MD, PhD; Anna But, MSc, PhD; Ivan Marinkovic MD, PhD; Jorge Rodríguez-Pardo, MD, PhD; Markku Kaste MD, PhD; Turgut Tatlisumak MD, PhD; Jukka Putaala MD, PhD

**Corresponding author**

Jenna Broman

Department of Neurology, Helsinki University Hospital

Haartmaninkatu 4, FI-00029, Helsinki, Finland.

Tel: +358 9 4711

E-mail: [jenna.broman@fimnet.fi](mailto:jenna.broman@fimnet.fi)

This Online Resource figure was created with SPSS 25.0 for Windows (SPSS Inc., IBM, Armonk, NY, USA)

**Online Resource 3** Kaplan-Meier curves for survival probabilities of initiating post-stroke antidepressants in patients using antidepressants at different time points before the index ischemic stroke.


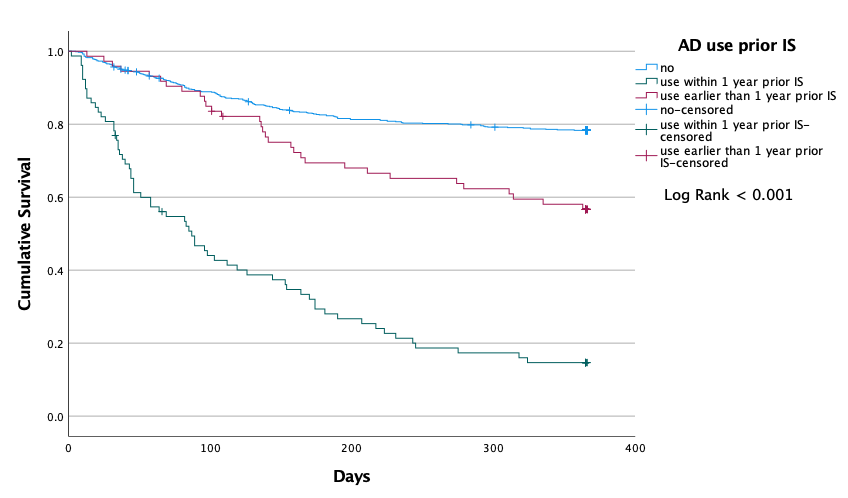


AD = antidepressant, IS = ischemic stroke
